# Uric Acid as a Predictor for Early Allograft Dysfunction after Living Donor Liver Transplantation: A Prospective Observational Study

**DOI:** 10.3390/jcm10122729

**Published:** 2021-06-21

**Authors:** Li-Min Hu, Hsin-I Tsai, Chao-Wei Lee, Hui-Ming Chen, Wei-Chen Lee, Huang-Ping Yu

**Affiliations:** 1 Department of Anesthesiology, Chang Gung Memorial Hospital, Linkou Branch, Taoyuan 333, Taiwan; mp1820@cgmh.org.tw (L.-M.H.); tsaic79@cgmh.org.tw (H.-I.T.); 2 College of Medicine, Chang Gung University, Taoyuan 333, Taiwan; alanlee@cgmh.org.tw (C.-W.L.); weichen@cgmh.org.tw (W.-C.L.); 3 Department of General Surgery, Chang Gung Memorial Hospital, Linkou Branch, Taoyuan 333, Taiwan; 4 Center for Big Data Analytics and Statistics, Chang Gung Memorial Hospital, Linkou Branch, Taoyuan 333, Taiwan; hmchen@cgmh.org.tw; 5 Department of Liver and Transplant Surgery, Chang Gung Memorial Hospital, Linkou Branch, Taoyuan 333, Taiwan

**Keywords:** liver transplantation, early allograft dysfunction, uric acid, reperfusion injury, oxidative stress, uric acid therapy, antioxidant

## Abstract

Early allograft dysfunction (EAD) is a postoperative complication that may cause graft failure and mortality after liver transplantation. The objective of this study was to examine whether the preoperative serum uric acid (SUA) level may predict EAD. We performed a prospective observational study, including 61 donor/recipient pairs who underwent living donor liver transplantation (LDLT). In the univariate and multivariate analysis, SUA ≤4.4 mg/dL was related to a five-fold (odds ratio (OR): 5.16, 95% confidence interval (CI): 1.41–18.83; OR: 5.39, 95% CI: 1.29–22.49, respectively) increased risk for EAD. A lower preoperative SUA was related to a higher incidence of and risk for EAD. Our study provides a new predictor for evaluating EAD and may exert a protective effect against EAD development.

## 1. Introduction

One of the curative treatments for end-stage liver disease is liver transplantation. As a result of the urgent need for liver transplantation and a critical lack of liver grafts from deceased donors, living donor liver transplantation (LDLT) became a solution to this plight in 1994 [1]. However, early allograft dysfunction (EAD) is a common complication leading to postoperative morbidity and mortality after liver transplantation [2,3]. EAD is the result of ischemia/reperfusion injury from reactive oxygen species following graft injury [4]. EAD patients are at greater risk for postoperative graft dysfunction or even graft failure that may require re-transplantation. That said, only a few of these patients undergo re-transplantation due to the scarce supply of matched liver donors. Donor- and recipient-related risk factors have been established in association with the development of EAD, such as donor body mass index (BMI), donor age, recipient age, cold ischemia time, and recipient liver mass [5,6], all of which should be taken into consideration before and during liver transplantation.

Recently, uric acid (UA) has been widely studied for its antioxidant capacity. UA is a terminal product of purine metabolism and a potent water-soluble molecule that accounts for more than half of the antioxidant capacity in human plasma [7,8]. UA can stabilize vitamin C in serum and eliminate peroxynitrite, which may result in nitric oxide donor formation in vitro [9]. Traditionally, UA is notorious for its relationship with gout, cardiovascular disease, and metabolic syndrome [7]. However, interestingly, a low serum concentration of UA was reported to be associated with a higher prevalence of and deterioration in some neurological diseases, including Alzheimer’s disease and Parkinson’s disease, as well as a worse outcome after acute ischemic stroke [10,11,12]. Increasing evidence has shown that UA plays a crucial role as an antioxidant and contributes to hepatic antioxidant capacity [13]. We hypothesized that higher preoperative serum uric acid (SUA) level may exert a protective effect against EAD.

## 2. Materials and Methods

### 2.1. Objectives

**Figure 1:**
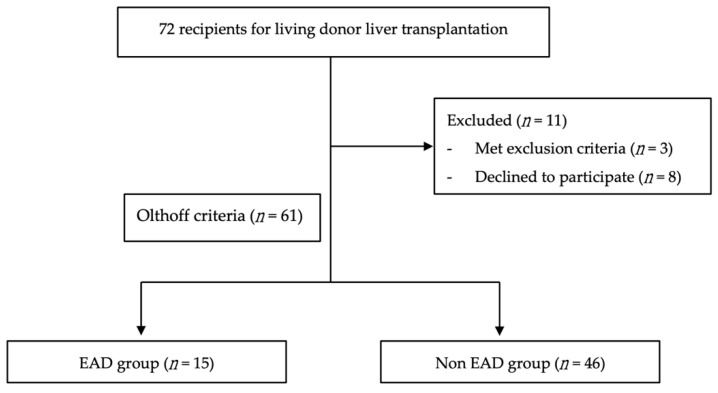
Flow diagram of patient selection and allocation. EAD, early allograft dysfunction.

This prospective, observational, single-center, hospital-based study was executed after receiving approvals from the Institutional Review Board of Chang Gung Memorial Hospital, Linkou Branch, Taiwan (Registration Numbers: 201800847A3, CMRPG3K1881, CMRPG3H1191, CMRPG3H11912 CMRPG3H1193). Septic patients, patients with shock, patients with preoperative pulmonary hypertension wedge pressure higher than 35 mmHg, and patients who declined to participate were excluded from the study. A total of 61 pairs of donors and recipients who performed LDLT at Taiwan Chang Gung Memorial Hospital (Taoyuan, Taiwan) from October 2018 to March 2020 were recruited into the study, as shown in Figure 1.

### 2.2. Data Collection and Variable Definition

In all cases, a biological model of the end-stage liver disease (MELD) score was measured on the day of the operation. All the patients were fasted at least 8 hours before liver transplantation. Before the induction of general anesthesia, blood samples were obtained from the arterial catheter and urine samples from the indwelling urinary catheter. Alanine aminotransferase (ALT), blood urea nitrogen (BUN), creatinine (Cre), SUA and urine uric acid (UUA) were measured in the hospital’s clinical laboratory. The estimated glomerular filtration rate (eGFR) was measured using the formula: eGFR (mL/min/1.73 m^2^) = 186 × Cre^−1.154^ × age^−0.203^ × (0.742 if women). Chronic kidney disease (CKD) was defined as a GFR less than 60 mL/min/1.73 m^2^. Cold ischemia time (CIT) was determined as the interval from the cold storage solution preservation to liver graft implantation. Warm ischemia time (WIT) was determined as the interval from hepatic vein reconstruction to portal vein reperfusion. The postoperative EAD was diagnosed according to the Otholff criteria as one or more of the following conditions: international normalized ratio ≥ 1.6 on Day 7, total bilirubin ≥ 10 mg/dL on Day 7, and alanine or aspartate aminotransferases ≥ 2000 IU/L within 7 days after liver transplantation [14].

### 2.3. Statistical Analysis

Established risk factors for EAD in this cohort were identified from previous reports [15,16]. Identified recipient-related risk factors were age, BMI, ALT, and MELD score. Donor-related risk factors were age, BMI, liver mass, and CIT. Numerical variables including MELD score, age, BMI, ALT, UUA, BUN, Cre, eGFR, graft size, graft recipient weight ratio (GRWR), intraoperative blood loss, CIT, and WIT were expressed as the mean and standard deviation. Categorical variables including SUA, blood type, sex, type of virology, and hepatocellular carcinoma (HCC) are shown as numbers and percentages. The association of the threshold SUA, comorbidities, demographic variables, and laboratory variables with the incidence of EAD was identified by univariate logistic regression analysis. With no selection criteria, the aforementioned potential risk factors of EAD following LDLT were then analyzed in the multiple logistic regression model to establish the independent influence of SUA on EAD. The strength of the association of each variable with EAD was summarized by calculating the odds ratio (OR) and corresponding 95% confidence interval (CI) from the coefficients estimated in the logistic regression models. Kaplan–Meier plots and Cox regression models were generated for survival analysis and prognostic factor analysis. All of the aforementioned data were analyzed using SAS 9.4 (SAS Institute Inc., Cary, NC, USA) statistical software. A *p*-value <0.05 was considered statistically significant.

## 3. Results

### 3.1. Baseline Characteristics

A total of 72 recipients were initially recruited to the study. After excluding three who met the exclusion criteria and eight who declined to participate, 61 recipients remained in this study. In this cohort, the mean age of the donors and recipients was 32.34 ± 9.21 and 55.13 ± 10.43 years, respectively. Among the recipients, 44 (72.13%) were males and 17 (27.86%) were females. Furthermore, 32 had hepatitis B virus (HBV)-related cirrhosis, 11 had hepatitis C virus (HCV)-related cirrhosis, 20 had alcoholic cirrhosis, and the remaining 27 had HCC. Intraoperatively, the graft size was, on average, 640 ± 131.43 g, with a GRWR of 0.98% ± 0.21%. Cold and warm ischemia times were on average 39.77 ± 25.97 and 16.41 ± 4.19 min, respectively. Additionally, 15 (24.59%) recipients developed EAD postoperatively and 11 died within 1 year of the operation. After evaluating the continuous relationship between the development of SUA and EAD, the SUA threshold was determined to be 4.4 mg/dL. Subjects were then divided into low SUA group (defined as ≤4.4 mg/dL; *n* = 27) and high SUA group (defined as >4.4 mg/dL; *n* = 34). The clinical characteristics of the patients are presented in Table 1 and Table 2.

### 3.2. SUA Level in Different Subgroups

We analyzed SUA levels in subgroups as shown in Table 3. In our study group, no patients had a history of gout in EAD group and thus no further analysis was conducted in regards to the effect of uric acid lowering drug for SUA level. Male recipients appeared to have lower UUA than female recipients. In patients who had alcoholism, chronic kidney disease (CKD), diabetes mellitus (DM) and hypertension (HTN), their UUA levels were lower. However, no statistical significance was observed in all aforementioned subgroups.

### 3.3. Risk Factors for Early Allograft Dysfunction

**Figure 2:**
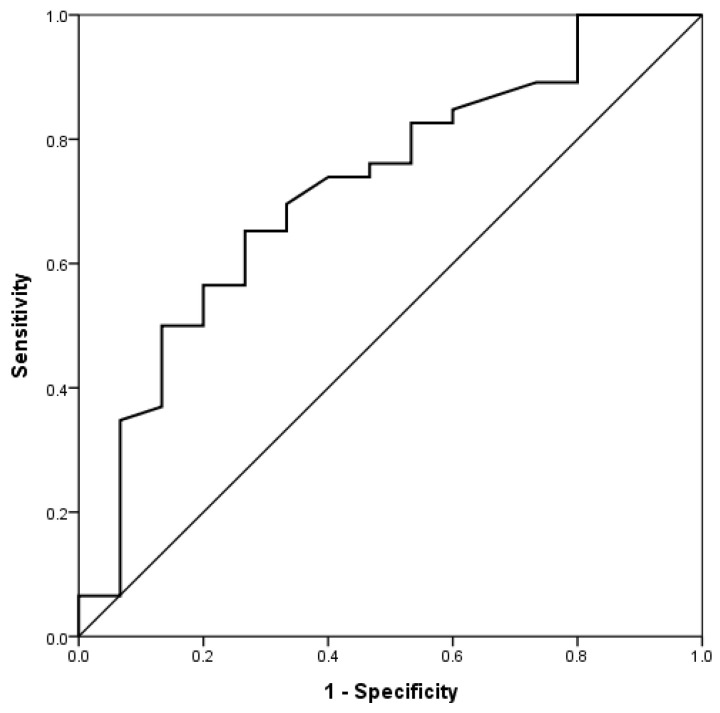
Receiver operating characteristic (ROC) analysis to determine the cutoff value of serum uric acid for the prediction of early allograft dysfunction.

The clinical definition of hyperuricemia corresponds to a urate concentration exceeding 7 mg/dL for men and 6.5 mg/dL for women. We aimed to investigate the optimal cutoff value of SUA with maximal odds ratio for the prediction of EAD. The optimal cutoff value using the receiver operating characteristic (ROC) result in our study with SUA ≤ 4.4 mg/dL was related to a maximal odds ratio, as observed in Figure 2. Furthermore, we use SUA as a categorical variable instead of continuous variable for further evaluation. In the univariate analysis, SUA ≤ 4.4 mg/dL was related to a five-fold (OR: 5.16, 95% CI: 1.41–18.83) increased risk for EAD. Similarly, in the multivariate analysis, SUA ≤ 4.4 mg/dL also was associated with a five-fold (OR: 5.39, 95% CI: 1.29–22.49) increased risk for EAD. Potential risk factors for EAD, including blood type O, SUA, and MELD score, were established on the basis of the results of the univariate analysis. All significant factors in the univariate analysis were included to create a multivariate logistic regression model. Only SUA remained as an independent prognostic factor in the multivariate logistic regression model (Table 4).

### 3.4. SUA and the Association with Early Allograft Dysfunction

**Figure 3:**
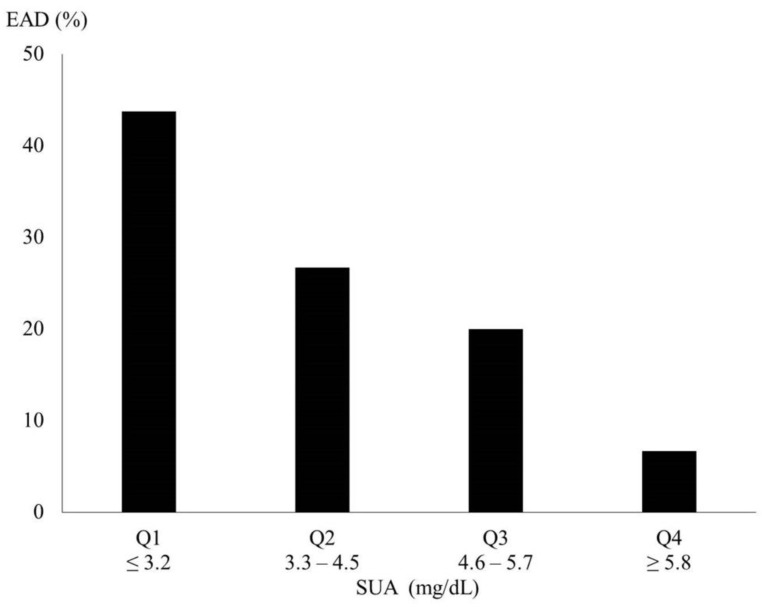
Incidence of EAD in association with the levels of preoperative SUA. SUA, serum uric acid; EAD, early allograft dysfunction.

To further demonstrate that lower preoperative SUA was associated with a higher risk for EAD, the SUA data were divided into quartiles. The incidences of EAD in association with SUA are shown in Figure 3. We demonstrate that a higher SUA was related to a lower incidence of EAD.

### 3.5. Early Allograft Dysfunction and One Year Mortality

**Figure 4:**
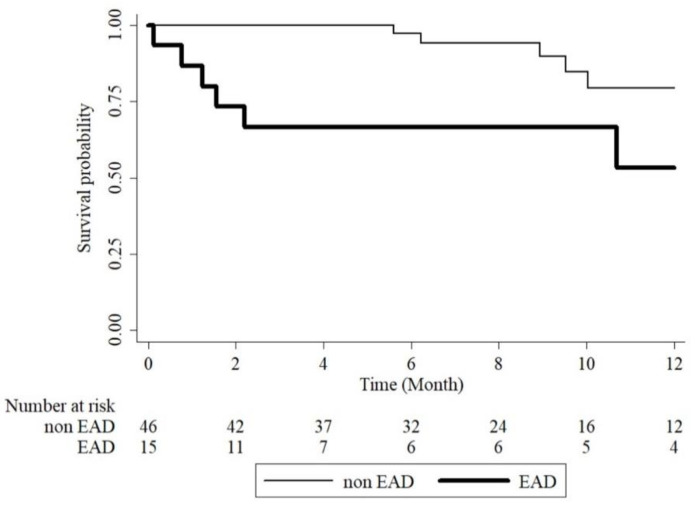
The one year survival curves of EAD versus non-EAD patients. EAD, early allograft dysfunction.

Kaplan–Meier survival curves showed that the EAD group had a lower survival proportion than the EAD group (log-rank test, *p* = 0.0006), as shown in Figure 4. When adjusting for several variables using a Cox proportional hazards model, the EAD group had a significantly higher hazard ratio (HR) than the non-EAD group (HR: 6.69, 95% CI: 1.66–26.92, *p* = 0.008), as shown in Table 5. We also noted the trend between increasing SUA related to decreasing mortality but not statistically significant.

## 4. Discussion

As a result of the urgent need for liver transplantation and a critical lack of grafts from donation after circulatory death or donation after brain death, living donor liver donation has become a solution to the organ shortage dilemma. However, EAD, with an incidence of 15–27%, is a common postoperative complication that may lead to morbidity and mortality after liver transplantation [3,5,17]. Some risk factors for the development of EAD, whether donor-, recipient-, or surgery-related, have been identified that may help transplant surgeons take precautions before or during liver transplantation. EAD is the result of ischemia/reperfusion injury from reactive oxygen species following graft injury. Oxidative stress is related to the gradual increase in reactive oxygen species and adversely affects the pathogenesis of ischemia/reperfusion injury. Thus, safe and effective therapeutic agents with antioxidant properties to mitigate oxidative damage may provide another different treatment strategy.

Serum UA has been recently studied for its antioxidant activity in various diseases. UA is a final breakdown product of purine nucleotides, and its metabolism involves factors that regulate both hepatic production and renal excretion. Initially, two purine nucleotides, adenine and guanine, are converted into inosine and guanosine, before being further converted into the purine bases hypoxanthine and guanine, respectively. Hypoxanthine and guanine are then oxidized and deaminated by hypoxanthine oxidase (XO) and guanine deaminase to form xanthine, which is again oxidized by XO to form UA [9]. Disturbances of production and excretion can lead to abnormal SUA levels whether hypouricemia or hyperuricemia. The liver is the major site of UA production. In severe hepatocellular injury, XO activity is reduced for the production of UA [18]. The kidneys are the major site for UA excretion. About 65–75% of UA produced daily is excreted via kidneys [19]. SUA levels are proved positively correlated to urinary excretion of uric acid [20]. Ischemia/reperfusion injury after liver transplantation is complex and involves various pathways such as the activation of Toll-like receptors (TLRs), changes in messenger RNA (mRNA) expression, the generation of reactive oxygen species (ROS), the regulation of autophagy, and the activation of hypoxia-inducible factors [21]. UA has shown a protective role in reducing TLR4/nuclear factor kappa B (NF-κB) activation, reducing ROS production, and regulating apoptosis [22,23,24]. Interestingly, in healthy humans, the acute elevation of UA seems to prevent the increase in oxidative stress and arterial stiffness caused by hyperoxia [25]. UA treatment was also reported to prevent the worsening of early ischemic injury related to reperfusion after acute stroke in patients receiving thrombolysis [26,27]. Thus, a low UA level may imply the severity of initial liver injury and susceptibility to later reperfusion injury following liver transplantation. 

In our study, we demonstrated that recipients with a lower preoperative SUA (≤4.4 mg/dL) are at a five-fold increased risk for the development of EAD postoperatively. Furthermore, those who developed EAD were at a six-fold increased risk of mortality, one year postoperatively. Such findings are exciting; however, limitations still apply to this study. Even though dietary habits may affect SUA level, all patients had SUA levels within normal range and maintained fasted for at least 8 hours preoperatively [28]. The small population size limited to Asian ethnicity and living donor transplantation patients can be a potential limitation, and validation in a large cohort including different ethnic groups is warranted. Even though SUA is an antioxidant, it is an approved risk factor for metabolic syndrome and cardiovascular complications [7,29]. The balance between risks and benefits can be challenging. Additional research is required to examine the altered enzymatic activities in the UA pathway in liver-diseased individuals and to validate the hypothesis that UA therapy may help in lowering the risk for EAD.

## 5. Conclusions

UA plays an important role in the human body due to its antioxidant capacity. We identified that preoperative SUA may be a potential biomarker to predict the development of EAD following LDLT. Higher preoperative SUA may exert a protective effect against EAD development.

## Figures and Tables

**Table 1:** Baseline characteristics of liver transplantation patients.

Characteristics	Recipient (*N* = 61)	Donor (*N* = 61)
Age (years)	55.13 ± 10.43	32.34 ± 9.21
MELD	16.67 ± 8.09	
BMI (kg/m^2^)	25.28 ± 4.27	23.00 ± 2.64
Preoperative ALT (U/L)	69.31 ± 149.85	
Preoperative SUA (mg/dL)	4.83 ± 2.58	
Preoperative UUA (mg/dL)	54.40 ± 59.65	
Preoperative BUN (mg/dL)	18.01 ± 11.78	
Preoperative Cre (mg/dL)	0.87 ± 0.49	
Preoperative eGFR (mL/min/1.73 m^2^)	113.69 ± 50.12	
Gender		
Male	44 (72.13)	33 (54.10)
Female	17 (27.87)	28 (45.90)
Blood type		
A	20 (32.79)	17 (27.87)
B	15 (24.59)	11 (18.03)
O	24 (39.34)	33 (54.10)
AB	2 (3.28)	0 (0)
ABO incompatibility	5 (8.20)	
Comorbidity		
HBV	32 (52.46)	
HCV	11 (18.03)	
Alcoholism	20 (32.79)	
HCC	27 (44.26)	
CKD	8 (13.11)	
DM	15 (24.59)	
HTN	21 (34.43)	
Gout	3 (4.92)	
Medication (Diuretics/ACEi/ARB)	42 (68.85)	
Intraoperative Parameters		
Graft size (g)	640.41 ± 131.43	
Blood loss (mL)	1977.38 ± 2181.03	
GRWR (%)	0.98 ± 0.21	
Cold ischemia time (min)	39.77 ± 25.97	
Warm ischemia time (min)	16.41 ± 4.19	
Outcomes		
EAD	15 (24.59)	
1 year mortality	11 (18.03)	

Data are presented as the mean ± SD or number and percentage in parenthesis. MELD, model of end-stage liver disease; BMI, body mass index; ALT, alanine aminotransferase; SUA, serum uric acid; BUN, blood urea nitrogen; UUA, urine uric acid; Cre, creatinine; eGFR, estimated glomerular filtration rate; HBV, hepatitis B virus; HCV, hepatitis C virus; HCC, hepatocellular carcinoma; CKD, chronic kidney disease; DM, diabetes mellitus; HTN, hypertension; ACEi, angiotensin converting enzyme inhibitor; ARB, angiotensin receptor blocker; GRWR, graft recipient weight ratio; EAD, early allograft dysfunction.

**Table 2:** A comparison of non-EAD and EAD patients.

Characteristics	Non-EAD (*N* = 46)	EAD (*N* = 15)	*p*-Value
Recipient			
Age (years)	56.37 ± 10.41	51.33 ± 9.88	0.105
MELD	15.20 ± 7.66	21.20 ± 7.92	0.011
BMI (kg/m^2^)	25.13 ± 4.10	25.71 ± 4.86	0.656
Sex			
Male	33 (71.74)	11 (73.33)	1.000
Female	13 (28.26)	4 (26.67)	
Blood type			
A	18 (39.13)	2 (13.33)	0.162
B	11 (23.91)	4 (26.67)	
O	15 (32.61)	9 (60.00)	
AB	2 (4.35)	0 (0.00)	
ABO incompatible	3 (6.52)	2 (13.33)	0.589
Preoperative ALT (U/L)	68.13 ± 169.1	72.93 ± 65.73)	0.874
Preoperative SUA (mg/dL)	5.26 ± 2.57	3.51 ± 2.19	0.021
Low UA (≤4.4 mg/dL)	16 (34.78)	11 (73.33)	0.009
High UA (>4.4 mg/dL)	30 (65.22)	4 (26.67)	
Preoperative UUA (mg/dL)	59.45 ± 66.80	38.93 ± 23.79	0.082
Preoperative BUN (mg/dL)	17.89 ± 11.92	18.37 ± 11.74	0.891
Preoperative Cre (mg/dL)	0.83 ± 0.46	0.98 ± 0.58	0.309
Preoperative eGFR (mL/min/1.73 m^2^)	114.61 ± 44.77	110.88 ± 65.68	0.805
Comorbidity			
HBV	25 (54.35)	7 (46.67)	0.605
HCV	7 (15.22)	4 (26.67)	0.439
Alcoholism	16 (34.78)	4 (26.67)	0.754
HCC	23 (50.00)	4 (26.67)	0.114
CKD	5 (10.87)	5 (33.33)	0.101
DM	10 (21.74)	5 (33.33)	0.491
HTN	16 (34.78)	5 (33.33)	0.918
Gout	3 (6.52)	0 (0.00)	0.569
Medication (Diuretics/ACEi/ARB)	32 (69.57)	10 (66.67)	0.833
Donor			
Age (years)	31.61 ± 9.13	34.60 ± 9.38	0.278
BMI (kg/m^2^)	22.87 ± 2.85	23.38 ± 1.87	0.521
Sex			
Male	25 (54.35)	8 (53.33)	0.945
Female	21 (45.65)	7 (46.67)	
Blood type			
A	15 (32.61)	2 (13.33)	0.345
B	8 (17.39)	3 (20.00)	
O	23 (50.00)	10 (66.67)	
AB	0 (0.00)	0 (0.00)	
Intraoperative Parameters			
Graft size (g)	635.50 ± 127.60	655.30 ± 146.20	0.617
Blood loss (mL)	1704.80 ± 2035.60	2813.30 ± 2464.80	0.087
GRWR (%)	0.97 ± 0.20	1.00 ± 0.27	0.588
Cold ischemia (min)	39.07 ± 25.4	41.93 ± 28.48	0.714
Warm ischemia (min)	16.07 ± 4.41	17.47 ± 3.31	0.264
Outcomes			
1 year mortality	5 (10.87)	6 (40.00)	0.019

Data are presented as the mean ± SD or number and percentage in parenthesis. MELD, model of end-stage liver disease; BMI, body mass index; ALT, alanine aminotransferase; SUA, serum uric acid; BUN, blood urea nitrogen; UUA, urine uric acid; Cre, creatinine; eGFR, estimated glomerular filtration rate; HBV, hepatitis B virus; HCV, hepatitis C virus; HCC, hepatocellular carcinoma; CKD, chronic kidney disease; DM, diabetes mellitus; HTN, hypertension; ACEi, angiotensin converting enzyme inhibitor; ARB, angiotensin receptor blocker; GRWR, graft recipient weight ratio; EAD, early allograft dysfunction.

**Table 3:** SUA levels in different patient groups.

Sex	Female (*N* = 17)	Male (*N* = 44)	*p*-Value
SUA (mg/dL)	4.81 ± 2.68	4.84 ± 2.57	0.960
UUA (mg/dL)	60.36 ± 108.11	52.10 ± 24.42	0.759
Alcoholism	Non alcoholism (*N* = 41)	Alcoholism (*N* = 20)	*p*-Value
SUA (mg/dL)	4.87 ± 2.73	4.77 ± 2.30	0.888
UUA (mg/dL)	56.62 ± 71.25	49.85 ± 22.74	0.582
CKD	Non-CKD (*N* = 51)	CKD (*N* = 10)	*p*-Value
SUA (mg/dL)	4.51 ± 2.13	6.48 ± 3.94	0.155
UUA (mg/dL)	58.02 ± 63.85	35.97 ± 24.94	0.073
DM	Non-DM (*N* = 46)	DM (*N* = 15)	*p*-Value
SUA (mg/dL)	4.56 ± 2.31	5.67 ± 3.21	0.151
UUA (mg/dL)	58.05 ± 66.91	43.323 ± 26.10	0.220
HTN	Non-HTN (*N* = 40)	HTN (*N* = 21)	*p*-Value
SUA (mg/dL)	5.01 ± 2.56	4.49 ± 2.65	0.458
UUA (mg/dL)	60.11 ± 71.65	43.54 ± 21.78	0.183

Data are presented as the mean ± SD in parenthesis. MELD, model of end-stage liver disease; BMI, body mass index; ALT, alanine aminotransferase; SUA, serum uric acid; BUN, blood urea nitrogen; UUA, urine uric acid; Cre, creatinine; eGFR, estimated glomerular filtration rate; HBV, hepatitis B virus; HCV, hepatitis C virus; HCC, hepatocellular carcinoma; CKD, chronic kidney disease; DM, diabetes mellitus; HTN, hypertension; ACEi, angiotensin converting enzyme inhibitor; ARB, angiotensin receptor blocker; GRWR, graft recipient weight ratio; EAD, early allograft dysfunction.

**Table 4:** Univariate analysis and logistic regression analysis.

Characteristics	Univariate Analysis		Multivariate Analysis (Stepwise Selection)	
	OR (95% CI)	*p*-Value	OR (95% CI)	*p*-Value
Recipient				
Age	0.96 (0.91–1.01)	0.117		
MELD	1.09 (1.02–1.18)	0.018		
BMI	1.03 (0.90–1.18)	0.650		
Sex				
Male	1.08 (0.29–4.02)	0.905		
Female	Ref			
Blood type				
A	Ref			
B	3.27 (0.51–20.93)	0.215		
O	5.40 (1.01–28.93)	0.049		
AB	–			
ABO incompatible	2.21 (0.33–14.65)	0.413		
Preoperative SUA				
Low UA (≤4.4 mg/dL)	5.16 (1.41–18.83)	0.013	5.39 (1.29–22.49)	0.021
High UA (>4.4 mg/dL)	Ref			
ALT	1.00 (1.00–1.00)	0.914		
Comorbidity				
HBV	0.74 (0.23–2.37)	0.606		
HCV	2.03 (0.50–8.21)	0.323		
Alcoholism	0.68 (0.19–2.49)	0.562		
HCC	0.36 (0.10–1.31)	0.122		
CKD	4.10 (0.99–16.95)	0.051		
DM	1.80 (0.50–6.49)	0.369		
HTN	0.94 (0.27–3.22)	0.918		
Gout	–	–		
Medication (Diuretics/ACEi/ARB)	0.88 (0.25–3.04)	0.833		
Donor				
Age	1.04 (0.97–1.10)	0.276		
BMI	1.08 (0.86–1.35)	0.514		
Sex				
Male	0.96 (0.30–3.09)	0.945		
Female	Ref			
Blood type				
A	Ref			
B	2.81 (0.39–20.46)	0.307		
O	3.26 (0.63–17.01)	0.161		
AB	–	–		
Intraoperative Parameters				
Graft	1.00 (1.00–1.01)	0.610		
Blood loss	1.00 (1.00–1.00)	0.096		
GRWR	2.12 (0.15–30.71)	0.582		
Cold ischemia time	1.00 (0.98–1.03)	0.709		
Warm ischemia time	1.08 (0.94–1.24)	0.264		

OR, odds ratio; CI, confidence interval; Ref, reference; MELD, model of end-stage liver disease; BMI, body mass index; ALT, alanine aminotransferase; SUA, serum uric acid; BUN, blood urea nitrogen; UUA, urine uric acid; Cre, creatinine; eGFR, estimated glomerular filtration rate; HBV, hepatitis B virus; HCV, hepatitis C virus; HCC, hepatocellular carcinoma; CKD, chronic kidney disease; DM, diabetes mellitus; HTN, hypertension; ACEi, angiotensin converting enzyme inhibitor; ARB, angiotensin receptor blocker; GRWR, graft recipient weight ratio; EAD, early allograft dysfunction.

**Table 5:** Cox regression.

Characteristics	Univariate Analysis		Multivariate Analysis (Stepwise Selection)	
	HR (95% CI)	*p*-Value	HR (95% CI)	*p*-Value
Recipient				
Age	1.05 (0.97–1.14)	0.217		
MELD	0.99 (0.92–1.08)	0.879		
BMI	0.88 (0.74–1.04)	0.129		
Sex				
Male	1.91 (0.41–8.86)	0.409		
Female	Ref			
Blood type				
A	Ref			
B	1.14 (0.16–8.14)	0.893		
O	3.78 (0.78–18.31)	0.098		
AB	–	0.995		
ABO incompatible	1.18 (0.15–9.23)	0.876		
Preoperative SUA				
Low UA (≤4.4 mg/dL)	1.18 (0.36–2.87)	0.785		
High UA (>4.4 mg/dL)	Ref			
ALT	0.99 (0.98–1.01)	0.451		
Comorbidity				
HBV	1.45 (0.42–4.96)	0.555		
HCV	0.62 (0.08–4.88)	0.617		
Alcoholism	0.49 (0.11–2.29)	0.367		
HCC	1.58 (0.48–5.51)	0.450		
CKD	1.56 (0.34–7.26)	0.567		
DM	1.33 (0.34–7.40)	0.563		
HTN	1.75 (0.53–5.74)	0.357		
Gout	2.91 (0.62–13.69)	0.177		
Medication (Diuretics/ACEi/ARB)	0.94 (0.28–3.22)	0.923		
EAD	4.4 (1.33–14.53)	0.015	6.69 (1.66–26.92)	0.008
Donor				
Age	1.00 (0.94–1.06)	0.962		
BMI	0.95 (0.76–1.20)	0.662		
Sex				
Male	1.84 (0.54–6.33)	0.332		
Female	Ref			
Blood type				
A	Ref			
B	1.07 (0.18–6.43)	0.939		
O	1.25 (0.31–5.01)	0.754		
AB	*–*	*–*		
Intraoperative Parameters				
Graft	1.00 (0.99–1.00)	0.253		
Blood loss	1.00 (1.00–1.00)	0.451		
GRWR	0.78 (0.06–10.98)	0.856		
Cold ischemia	1.00 (0.98–1.02)	0.961		
Warm ischemia	0.93 (0.80–1.07)	0.292		

HR, hazard ratio; CI, confidence interval; Ref, reference; MELD, model of end-stage liver disease; BMI, body mass index; ALT, alanine aminotransferase; SUA, serum uric acid; HBV, hepatitis B virus; HCV, hepatitis C virus; HCC, hepatocellular carcinoma; CKD, chronic kidney disease; DM, diabetes mellitus; HTN, hypertension; ACEi, angiotensin converting enzyme inhibitor; ARB, angiotensin receptor blocker; GRWR, graft recipient weight ratio; EAD, early allograft dysfunction.

## Data Availability

The data presented in this study are available on request from the corresponding author. The data are not publicly available.
